# What to Do in Emergencies

**Published:** 1872-01

**Authors:** 


### WHAT TO DO IN EMERGENCIES.

If a person falls down in a fit, and begins to snore loudly, with very red face, it is apoplexy; let him be seated so as to favor the blood going downwards, away from the head; apply cold cloths to the head, or cushions of equal quantities of snow or pounded ice and common salt. If the person is perfectly still, face pale, and there is no perceptible breathing, it is a fit of

### FAINTING.

Do not touch him, except to loosen the clothing; then keep off five or ten feet distant, so as to allow the air to come in; make no noise, and there will very soon be a calm, quiet return to consciousness and life; for it is only a momentary cessation of the circulation of the blood to the head. But suppose there is a very violent motion of hands and feet, and all sorts of bodily contortions, it is

### EPILEPSY.

Let the man contort until he is tired; you can't hold him still; all your efforts only tend to aggravate the trouble and to exhaust the strength; all that ought to be done is to keep the unfortunate from hurting himself. There is no felt suffering, for as soon as he comes to he will tell you that he remembers nothing whatever of what has passed, appears to be the only calm and self-possessed person in the whole crowd, and is apparently as perfectly well as before the occurrence.

### DIZZINESS

Often comes instantaneously, and we begin to reel before we know it. Shut the eyes, whether you are walking along the street, looking over a precipice, ascending a ladder, or climbing to a ship's masthead; the fear or dizziness disappears instantly if you look upward. Suppose a person comes at you in

A TEARING PASSION, gesticulating wildly, and speaking loud enough to wake the dead. Don't be afraid; such people are not dangerous; barking dogs don't bite, nor hissing geese. Wait until the man exhausts himself, and a minute longer, keeping upon him a calm and fearless eye; then let your words be few and quiet as possible; be forbearing, conciliating, but as firm as the rocks of the Andes. That is the best way to annihilate your adversary; and he will respect and fear you till life's latest hour.

PRESENCE OF MIND

Is one of the most valuable traits of human character in the conduct of life; it always gives a man the vantage ground as to others, and is the way of safety in all conditions of human peril. In a steamboat collision at midnight, the passengers bounced out of their berths, grabbed their life-preservers, and began to adjust them with all the rapidity possible; being of India rubber, they needed to be inflated; among them all was a very fat old woman; she tried to tie it around her, but it would not meet. She then endeavored to inflate it, but could not find the pipe; and in her desperation she ran screaming through the cabin, vociferously calling upon every one she met,

"BLOW ME UP."

This was a want of presence of mind, as it was in the case of an old bach, on retiring after having been at a party where beauty ruled the hour; he became so absorbed in the reminiscences of the past, that he put his pantaloons to bed, and hung himself on the back of a chair; so with the young lady who took a letter to the post-office for a nice young man, far away, and put herself in the box instead of the letter.

A gentleman on a visit to a Lunatic Asylum then at Staunton, Virginia, was looking at the beautiful scenery of hill and valley from the parapet, when a man joined him, and entered into a pleasant conversation. "By the way," said he, "let us jump down here." "Anybody can do that," said the visitor, "but I can go down and jump up here; come and see;" and with that led the way to the bottom of the building, the other following, who was given into the custody of the first keeper met on the way.

The visitor saw at once that it was one of the escaped lunatics, and feeling confident that it would do no good, but would rather enrage the man to enter into a controversy, determined, in a moment, to humor him, to throw him off his guard, and excite his curiosity.

On another occasion, at a gentleman's country seat, a son was spending a few days, and not being considered in his right mind he had a keeper, whom he frequently eluded. On one of these occasions he went into the drawing-room where was his mother, alone, in full dress, waiting for company; a fine old English lady she was. "Mother," said he, closing the door adroitly and locking it, "I am going to cut your throat with this razor." "Why, my son, the blood would spoil my beautiful white dress; let me go and change it first;" and rising from her seat, she went towards the door in a most assured manner, thus not only concealing all fear, but appealing so strongly to his own consciousness of the truth of her statement, that it threw him off his guard, and he opened the door without the slightest hesitation.

### A LADY.

And gentleman of this city had retired late; the husband fell into a sound sleep immediately; she remained awake for some time, and hearing a noise, as from lighting a match, she saw by the very dim light in the room that two men were there, one opening the drawers, the other making examinations elsewhere. In times of great peril the mind will run through an argument or a course of reasoning with lightning rapidity; hers reasoned thus: "If I wake my husband, he will rush upon the robbers; he without any weapon, they well armed, two against one; he half confused, they composed and fully understanding the situation, because fully awake; to disturb him would be almost certain death, to perhaps both of us; or if I let them know that I am awake." So she determined to remain as still as death, yet keep an eye upon them; they ransacked all the drawers, all the clothing at the bedside, and every cranny in the room where it was at all likely that any valuables could be found, gathering up money, gold, trinkets, watches, chains, diamonds, everything, to a large amount; but what were these to her husband's life? Not until they had left the room and had time to get into the street, did she wake up the loved sleeper at her side. And, man like, when told that there were burglars in the house, he scouted the idea with a "Tush!" and turned over to go to sleep again. But

SHE WOULDN'T HAVE IT SO,

And kept tugging at him until he got out of bed; and when he found that his pocket-book was gone, that his watch was nowhere, and that his "rib's" jewelry was in the same place, he waked up to the conviction that his wife was right, as sensible husbands generally do. So much for presence of mind, for calmness and wisdom in times of great emergency; a faculty of great value in the conduct of human life, and liable to be called into requisition in all places at all seasons of the year, and at any hour of the day or night; and they are wisest who cultivate it in hours of calmness, so that it shall be made available in tumult and in storm.

